# Successful Management of a Patient with Intraoperative Bleeding of More than 80,000 mL and Usefulness of QTc Monitoring for Calcium Correction

**DOI:** 10.1155/2021/6635696

**Published:** 2021-04-15

**Authors:** Yuki Sugiyama, Kazuma Aiba, Nariaki Arai, Mariko Ito, Masatoshi Urasawa, Chie Hirose, Ikuko Murakami, Ryusuke Tanaka, Tomokatsu Yamada, Keisuke Iida, Hiroyuki Nakamura, Mikito Kawamata

**Affiliations:** Department of Anesthesiology and Resuscitology, Shinshu University School of Medicine, Matsumoto, Japan

## Abstract

Intraoperative massive bleeding is associated with high rates of mortality and anesthetic management of massive bleeding is challenging because it is necessary to achieve volume resuscitation and electrolyte correction simultaneously during massive transfusion. We report a case of life-threatening bleeding of more than 80,000 mL during liver transplantation in which real-time QTc monitoring was useful for an extremely large amount of calcium administration for treatment of hypocalcemia. A 47-year-old female with a giant liver due to polycystic liver disease was scheduled to undergo liver transplantation. During surgery, life-threatening massive bleeding occurred. The maximum rate of blood loss was approximately 15,000 mL/hr and the total amount of estimated blood loss was 81,600 mL. It was extremely difficult to maintain blood pressure and a risk of cardiac arrest continued due to hypotension. In addition, even though administration of insulin and calcium was performed, electrolyte disturbances of hyperkalemia and hypocalcemia with prolongation of QTc interval occurred. At that time, we visually noticed that the QT interval was shortened in response to bolus calcium administration, and we used the change of real-time QTc interval as a supportive indicator for calcium correction. This monitoring allowed for us to administer calcium at an unusually high rate, by which progression of hypocalcemia was prevented. Levels of hemoglobin and coagulation factors were preserved both by restriction of crystalloid infusion and by a massive transfusion protocol. The patient was extubated without pulmonary edema or cardiac overload and was finally discharged without any sequelae. Intensive and cooperative management for massive transfusion and electrolyte correction using QTc monitoring was considered to be a key for successful management.

## 1. Introduction

Intraoperative massive bleeding sometimes occurs unexpectedly and is associated with high rates of morbidity and mortality [1, 2]. It has been reported that perioperative cardiac arrest due to electrolyte disturbances caused by blood transfusion during massive bleeding was accompanied by both hyperkalemia and hypocalcemia [3], and it is necessary to achieve volume resuscitation and electrolyte correction simultaneously during massive transfusion. We report a case of life-threatening bleeding of more than 80,000 mL during liver transplantation in which hyperkalemia and hypocalcemia occurred by massive transfusion and in which real-time QTc monitoring was useful for an extremely large amount of calcium administration for treatment of hypocalcemia. Written informed consent was obtained from the patient for publication of this case report and accompanying images.

## 2. Case Presentation

A 47-year-old female (height, 160 cm; weight, 60 kg) with a giant liver due to polycystic liver disease (PLD) was scheduled to undergo ABO-incompatible living donor liver transplantation because of worsening malnutrition, disability in activities of daily living, and abdominal distention, which were converted to 16 points by the model for end-stage liver disease score. In the past 3 years, coil embolization of the hepatic artery and percutaneous drainage of liver cysts had been performed. After the liver cyst drainage, biliary peritonitis occurred and she was treated with antibiotics.

**Figure 1:**
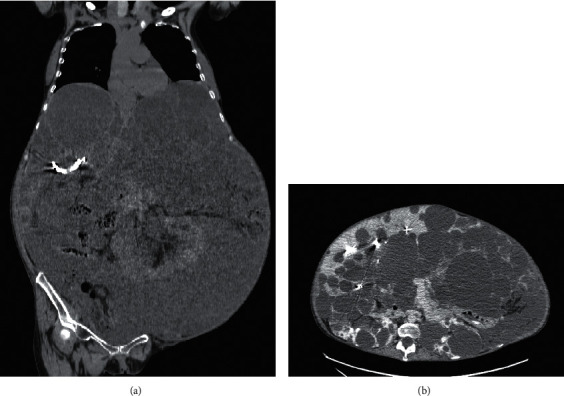
Preoperative images of computed tomography showing a massively enlarged polycystic liver that occupied the abdominal space and compressed the diaphragm and abdominal wall. (a) Coronal section. (b) Axial section.

Preoperative computed tomography revealed remarkable abdominal distension caused by an enlarged polycystic liver (Figure 1). Results of laboratory tests were as follows: hemoglobin (Hb), 10.1 g/dL; platelet (Plt) count, 27.4 × 10^4^/*μ*L; albumin, 2.2 g/dL; blood urea nitrogen, 31.3 mg/dL; creatinine, 0.90 mg/dL; total bilirubin, 0.31 mg/dL; alanine transaminase, 13 U/L; aspartate transaminase, 15 U/L; prothrombin time-international normalized ratio (PT-INR), 1.18; activated partial thromboplastin time (APTT), 30.0 sec; fibrinogen, 573 mg/dL. An electrocardiogram (ECG) showed normal sinus rhythm, and transthoracic echocardiography showed normal cardiac function. Portal hypertension was not predicted. The surgeons pointed out a risk of bleeding because of surgical difficulties due to PLD and strong adhesion induced by peritonitis. Blood loss anticipated by the surgeon was 15000 ml. The transfusion unit was preoperatively informed about the possibility of massive transfusion. We ordered 5,600 mL of O type red blood cells (RBC), 4,800 mL of AB type fresh frozen plasma (FFP), and 800 mL of AB type platelet concentrate (PC).

**Figure 2:**
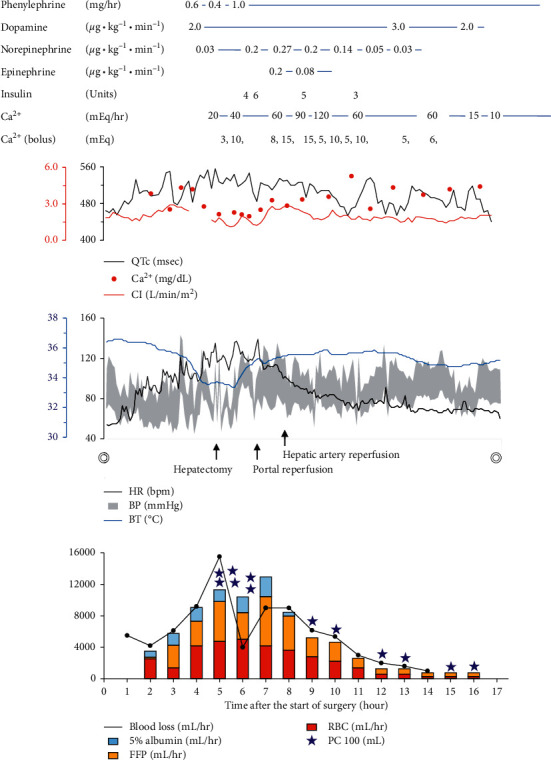
Overview of the anesthetic chart. The dosages of drugs are shown in the upper part. Serial changes in QTc interval (QTc), Ca^2+^, cardiac index (CI), hemodynamics, and body temperature (BT) are shown in the middle part. Approximate amounts of blood loss and transfusion (mL/hr) are shown in the bottom part. Time indicates time after the start of surgery. The double circles indicate the start and end of the surgery. HR, heart rate; bpm, beats per minute; BP, blood pressure; FFP, fresh frozen plasma; RBC, red blood cell concentrates; PC, platelet concentrates.

After induction of general anesthesia, three 18-gauge peripheral catheters, a 12-gauge triple-lumen central venous catheter, and a pulmonary arterial catheter were placed. Since total clamping of the inferior vena cava requiring veno-veno bypass might be necessary, two 4-Fr introducer sheaths were placed at the femoral vein and the intrajugular vein. Soon after the start of surgery, massive bleeding of more than 4,000 mL/hr occurred (Figure 2). We started massive transfusion of FFP at a ratio of about 1 : 1 with RBC. We also transfused 5% albumin and restricted the crystalloid solution. Reinfusion of salvaged blood and use of veno-veno bypass with retrieved blood from the operative field were avoided because many cysts of the PLD ruptured during surgery and the blood in the operative field was contaminated by the cystic content. We constantly ordered blood products and communicated with the transfusion unit.

**Figure 3:**
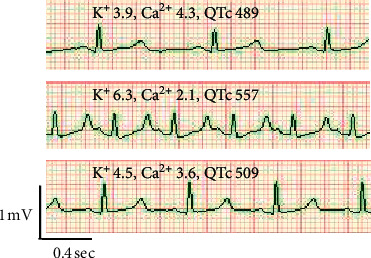
Changes in the electrocardiogram due to electrolyte abnormalities. Upper part: one hour after the start of surgery. Middle part: five hours after the start of surgery. Bottom part: nine hours after the start of surgery. The unit of K^+^ is mEq/L, the unit of Ca^2+^ is mg/dL, and the unit of QTc is msec.

The bleeding rate increased as the surgery progressed because bleeding occurred from the area where the tissue was detached from adhesion. Body temperature rapidly decreased despite the use of a rapid infusion and fluid warming system (Level 1 Fast Flow Fluid Warmer, Smiths Medical ASD Inc., Rockland, MA, USA) through 4-Fr introducer sheaths. Although catecholamine administration and massive transfusion from 5 venous lines including the introducer sheath were performed by many anesthesiologists, it was extremely difficult to maintain blood pressure and a critical situation with a risk of cardiac arrest continued due to hypotension. In addition, even though administration of insulin and calcium was performed, electrolyte disturbances of K^+^ of 6.3 mEq/L and Ca^2+^ of 2.1 mg/dL (normal range: 4.6–5.3 mg/dL) with peaked *T* wave and prolongation of QTc interval (557 msec) occurred (Figures 2 and 3). At that time, we visually noticed that the QT interval was shortened in response to bolus calcium administration, and we therefore displayed the QTc interval on a bedside monitor (CSM-1000 series Life Scope *G*, Nihon Kohden, Tokyo, Japan) and used the change of real-time QTc interval as a supportive indicator for calcium correction (maximum rate: 120 mEq/hr) combined with blood gas analysis. The hepatectomy was performed without veno-veno bypass 4.5 hours after the start of surgery and the bleeding rate at that time was 15,000 mL/hr (Figure 2). The excised polycystic liver weighed 10.0 kg.

Until portal reperfusion, we continued massive transfusion (approximately 12,000 mL/hr) including 300 mL of PC and sodium bicarbonate with additional fluid warming systems. After portal reperfusion, the bleeding rate gradually decreased and the hemodynamic variables and electrolyte disturbance improved (Figures 2 and 3; Table 1). After anastomosis of the hepatic artery, 1 g of tranexamic acid was administered. Except for PC, which was transported from transfusion centers in our region and/or neighboring regions, the supply of blood products was not interrupted and coagulation factors were maintained throughout the surgery by administration of FFP and 3 g of fibrinogen with at least 1.26 of PT-INR, 43.6 sec of APTT, and 116 mg/dL of fibrinogen (Table 1). The abdomen was packed with gauzes and closed because complete hemostasis was difficult. The patient was transferred to the intensive care unit (ICU). Soon after arrival at the ICU, she responded to command. On postoperative day (POD) 2, second-look surgery was performed for removal of packed gauzes. She was extubated on POD 3 without pulmonary edema or cardiac overload.

**Figure 4:**
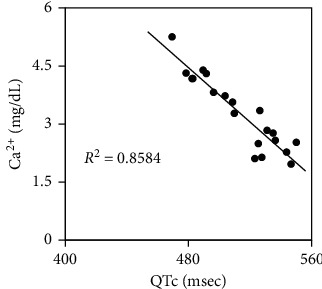
Correlation between Ca^2+^ and QTc interval. R^2^ indicates coefficient of determination.

The duration of surgery was 16 hours and 53 minutes and the duration of general anesthesia was 22 hours and 27 minutes. Throughout the surgery, anesthesia was maintained with 1.5% sevoflurane, 0.16–0.33 *μ*g^−1^ ・kg^−1^ ・min^−1^ of remifentanil, and intermittent fentanyl administration (total of 1.5 mg). Bispectral Index^TM^ (Aspect Medical Systems, Norwood, MA, USA) values were between 30 and 60. Cardiac index was between 1.2 and 2.8 L^−1^ ・min^−1^ ・m^−2^. A total of 742 mEq of calcium ion, 750 mL of 7% sodium bicarbonate, and 18 units of insulin were administered. Postoperative analysis of the relationship between QTc interval and Ca^2+^ during surgery showed a strong negative correlation (Figure 4). Representative laboratory data are shown in Table 1. Total infusion volume was 88,560 mL (crystalloid solution: 3,450 mL; 6% hydroxyethyl starch: 3,500 mL; 5% albumin: 10,490 mL; RBC: 34,160 mL; FFP: 34,600 mL; PC: 2,400 mL), and total output volume was 86,350 mL (estimated volume of blood loss including ascites and cystic fluid: 81,600 mL; urine output: 4,750 mL); therefore, intraoperative fluid balance was +2,210 mL. Although she suffered from acute rejection and sepsis, she finally regained independent living by rehabilitation and was discharged on POD 156.

## 3. Discussion

Although liver transplantation for PLD is still controversial, malnutrition and disability in activities of daily living have been common indicators for liver transplantation [4]. Severity of liver disease, coagulopathy, portal hypertension, and surgical factors has been identified as intraoperative risk factors for bleeding in patients undergoing liver transplantation [5–7]. Among these risk factors, coagulopathy and portal hypertension were not present in our patient. However, there is a critical perioperative risk for bleeding in patients with PLD because of surgical difficulties due to the enlarged liver [8, 9]. In addition, strong adhesion after peritonitis increases the risk of bleeding.

In the present case, transfusion management became extremely difficult, especially for dealing with hyperkalemia, hypocalcemia, and hypothermia. It is known that when the blood transfusion rate exceeds 100 to 150 mL/min, as in our case, hyperkalemia associated with blood transfusion tends to occur [10]. A previous study in which cardiac arrest due to hyperkalemia caused by intraoperative blood transfusion was investigated showed that almost all patients had hypothermia, acidosis, and hypocalcemia at the time of cardiac arrest [3].

Hypocalcemia during massive transfusion leads to coagulopathy and is associated with mortality [11, 12]. In this case, an unusually high rate of calcium administration up to 120 mEq/hr with additional 5–15 mEq bolus administration was required for correction of hypocalcemia and resulted in a total amount of 742 mEq of calcium administration during surgery. In fact, since there was a risk of ventricular arrhythmia and/or atrioventricular nodal block induced by overcorrection of calcium, we initially hesitated to administer such a high dose of calcium that we had not experienced. Fortunately, we noticed the relationship between Ca^2+^ and QTc interval during surgery and we could increase the amount of calcium up to 120 mEq/hr without concern of overcorrection. Actually, as shown in Figure 4, the QTc interval was strongly correlated with Ca^2+^.

The utility of continuous QTc monitoring has been studied in the fields of neonatal sleep apnea, neonatal development, and critical care patients' mortality [13–15]; however, its utility for calcium correction during bleeding has not been reported. Although we need to pay attention to many intraoperative factors that influence the QTc interval such as volatile anesthetics, antiarrhythmic agents, and hypokalemia, our case demonstrated that real-time QTc monitoring has a potential to be a useful monitor for calcium correction when bleeding is excessively massive and rapid.

Hypocalcemia also causes a decrease of cardiac contractility. As for a correlation between cardiac output and Ca^2+^, although we sometimes felt that there was a prompt improvement of hemodynamics by calcium administration, cardiac output data obtained from pulmonary artery catheter monitoring did not clearly correlate to Ca^2+^ (Figure 2). This was considered to be due to the influence of intraoperative changes in many factors that affect cardiac output such as circulating blood volume, compression of vessels by a surgical procedure, and amount of catecholamines.

To the best of our knowledge, this is the most severe intraoperative bleeding that has been reported in a patient who recovered and regained independent living. During massive bleeding, although we considered temporary suspension of surgery with gauze packing, since this operation was a living donor liver transplantation and the donor's liver operation had already started, the transplantation surgery was continued as much as possible. We administered tranexamic acid just after hepatic artery reperfusion because it has been reported that hyperfibrinolysis developed after reperfusion in living donor liver transplantation [16] and that hyperfibrinolysis was related to thrombosis in the portal vein and hepatic artery [17]. It might have been better to perform thromboelastography at some time points in this case. Further study is needed to determine the optimal dose and timing of administration of tranexamic acid in living donor liver transplantation. As in this case, even if disastrous bleeding occurs unexpectedly, the patient may recover with a massive transfusion protocol, electrolyte correction, and body temperature correction without intraoperative cardiac arrest and without any sequelae. We hope that our experience can help anesthesiologists when they encounter severe intraoperative massive bleeding.

## 4. Conclusions

We successfully managed a patient with PLD in whom life-threatening intraoperative bleeding of more than 80,000 mL occurred when performing liver transplantation. Intensive and cooperative management for massive transfusion and electrolyte correction using QTc monitoring is considered to be a key to successful management during intraoperative massive bleeding.

## Figures and Tables

**Table 1:** Representative laboratory data.

	Normal range	Time (hr:min)						
		Pre	3:30	5:10	6:50	10:00	14:00	16:40
FiO_2_		0.4	0.4	0.6	0.4	0.4	0.4	0.4
pH	7.35–7.45	7.53	7.32	7.16	7.28	7.35	7.40	7.42
PaCO_2_ (mmHg)	35.0–45.0	33.8	40.8	39.7	42.7	34.0	36.3	38.5
HCO_3_^−^ (mEq/L)	22–28	28.3	20.2	13.6	19.6	18.4	21.8	24.2
PaO_2_ (mmHg)		171	189	240	186	200	193	197
BE (mEq/L)	−2.4–+2.3	5.7	−5.1	−14.0	−6.1	−6.1	−2.1	0.2
Ca^2+^ (mg/dL)	4.6–5.3	4.4	4.3	2.1	2.5	3.6	3.7	4.4
Na^+^ (mEq/L)	135–145	139	138	138	148	148	148	149
K^+^ (mEq/L)	3.6–4.8	4.0	5.5	6.3	4.6	4.5	4.4	4.4
Glu (mg/dL)	73–140	94	195	232	244	232	201	171
Lac (mg/dL)	4–16	7	24	48	46	43	33	30
Hb (g/dL)	12–16	9.3	8.5	10.4	8.0	7.7	10.8	9.4
Plt (×10^4^/*μ*L)	15–35	23.7	4.8	0.8	10.8	3.9	7.0	10.2
PT-INR	0.85–1.15	1.28		1.17	1.26	1.14		1.09
APTT (second)	23–38	33.0		32.7	43.6	32.2		27.5
FIBG (mg/dL)	180–350	367		163	116	228		233
Blood loss (mL/hr)			6,120	15,500	9,000	6,150	1,000	0

Time indicates time after the start of surgery. Blood loss indicates approximate amount of blood loss per hour. Pre indicates preoperative data. FiO_2_, fraction of inspiratory oxygen; PaCO_2_, partial pressure of carbon dioxide; PaO_2_, partial pressure of arterial oxygen; BE, base excess; Glu, glucose; Hb, hemoglobin, Lac, lactate; Plt, platelet; PT-INR, prothrombin time-international normalized ratio; APTT, activated partial thromboplastin time; FIBG, fibrinogen.

## Data Availability

The data used to support the findings of this study are available from the corresponding author upon request.

## Abbreviations

APTT: Activated partial thromboplastin time
BE: Base excess
bpm: Beats per minute
BP: Blood pressure
BT: Body temperature
CI: Cardiac index
ECG: Electrocardiogram
FIBG: Fibrinogen
FFP: Fresh frozen plasma
FiO2: Fraction of inspiratory oxygen
Glu: Glucose
Hb: Hemoglobin
HR: Heart rate
Lac: Lactate
ICU: Intensive care unit
PC: Platelet concentrates
PLD: Polycystic liver disease
Plt: Platelet
POD: Postoperative day
Pre: Preoperative data
PT-INR: Prothrombin time-international normalized ratio
PaO_2_: Partial pressure of arterial oxygen
PaCO2: Partial pressure of carbon dioxide
QTc: QTc interval
RBC: Red blood cell concentrates
Time: Time after the start of surgery.
